# Prophylactic antibiotics for preventing ventilator-associated pneumonia: a pairwise and Bayesian network meta-analysis

**DOI:** 10.1186/s40001-023-01323-z

**Published:** 2023-09-15

**Authors:** Shanshan Zha, Jianyi Niu, Zhenfeng He, Wei Fu, Qiaoyun Huang, Lili Guan, Luqian Zhou, Rongchang Chen

**Affiliations:** 1 Guangzhou Institute of Respiratory Health, State Key Laboratory of Respiratory Disease, National Clinical Research Center for Respiratory Disease, National Center for Respiratory Medicine, First Affiliated Hospital of Guangzhou Medical University, 151 Yanjiang Road, Guangzhou, 510120 Guangdong China; 2 Respiratory Mechanics Laboratory, Guangzhou Institute of Respiratory Health, National Center for Respiratory Medicine, First Affiliated Hospital of Guangzhou Medical University, Guangzhou, China; 3 Department of Respiratory and Critical Care Medicine, Shenzhen Institute of Respiratory Diseases, Shenzhen People’s Hospital, First Affiliated Hospital of Southern University of Science and Technology, Second Clinical Medical College of Jinan University, Shenzhen, 518020 Guangdong China

**Keywords:** Prophylactic antibiotics, Ventilator-associated pneumonia, Network meta-analysis

## Abstract

**Background:**

The role of prophylactic antibiotics in preventing ventilator-associated pneumonia (VAP) in patients undergoing invasive mechanical ventilation (IMV) remains unclear. This network meta-analysis compared the efficacy and safety of antibiotic prophylaxis in preventing VAP in an IMV population in intensive-care units (ICUs).

**Methods:**

We searched the PubMed, Web of Science, Embase, and Cochrane Library databases from inception to December 2021, to identify relevant studies assessing the impact of prophylactic antibiotics on the incidence of VAP, the mortality, and the duration of ICU stays and hospitalization to perform a meta-analysis.

**Results:**

Thirteen studies (2144 patients) were included, 12 of which were selected for the primary analysis, which revealed that treatment with prophylactic antibiotics resulted in a lower VAP rate compared with control groups [risk ratio (RR) = 0.62]. Bayesian network meta-analysis indicated that aerosolized tobramycin and intravenous ampicillin–sulbactam presented the greatest likelihood being the most efficient regimen for reducing VAP.

**Conclusions:**

Antibiotic prophylaxis may reduce the incidence of VAP, but not the mortality, for adult patients undergoing IMV in ICUs. Tobramycin via nebulization and ampicillin–sulbactam via intravenous administration presented the greatest likelihood of being the most efficient regimen for preventing VAP. However, well-designed randomized studies are warranted before definite recommendations can be made.

**Supplementary Information:**

The online version contains supplementary material available at 10.1186/s40001-023-01323-z.

## Background

Despite advances in the understanding of the contributing causes, ventilator-associated pneumonia (VAP) continues to be a frequent complication in patients receiving invasive mechanical ventilation (IMV) in intensive-care units (ICUs). Published studies have reported a prevalence of VAP ranging from 5 to 40% in IMV patients in ICUs, with mortality estimated at 13–25.2% [1]. In addition, compared with similar patients without VAP, it has been demonstrated that VAP was associated with longer duration of IMV, longer hospital stays, and higher costs [2].

Several strategies have been put forward for preventing VAP, including regular oral care with chlorhexidine, prophylactic probiotics, prophylactic antibiotics, and using silver-coated endotracheal tubes [3–5]. Among these, the use of prophylactic antibiotics, with a research history of more than 30 years, is a subject of substantial debate. On the one hand, the use of prophylactic antibiotics may be the most efficient measure to directly kill many potential pathogenic bacteria associated with VAP. On the other hand, there is not enough evidence to affirm the efficacy of prophylactic antibiotics and, moreover, the use of prophylactic antibiotics may contribute to the emergence of multi-drug-resistant organisms and cause adverse events, such as nephrotoxicity and bronchospasm [4]. Controversy persists, and thus, the American Thoracic Society guidelines do not currently recommend the administration of antibiotic prophylaxis as a conventional treatment for VAP.

A meta-analysis of the use of preventive antibiotics, published in 2018, provided evidence of the protective effect of antibiotics against VAP [6]. However, that study only focused on antibiotic administration via the respiratory tract. Prophylaxis through the intravenous administration of antibiotics is an essential and inescapable part of clinical practice and must be considered when assessing the possible benefits and adverse impacts of prophylaxis. Furthermore, no recommendations regarding the choice of antibiotic, dose selection, and administration route have been made previously; thus, there is a need for further investigation to clarify the optimal administration route, antibiotic type, and dose. However, the application of traditional pairwise meta-analysis, using only a direct-comparison model, has not been able to address the aforementioned issues. Network meta-analysis (NMA), which enables comprehensive assessment from direct and indirect comparisons, is a useful method for generating a comprehensive view of the available evidence [7]. Therefore, the aim of the present study was to evaluate the effect of prophylactic antibiotics for preventing VAP in patients undergoing IMV. Furthermore, we used an NMA model to investigate the relative efficacy and safety of different administration routes and antibiotic types.

## Methods

This systematic review and meta-analysis were performed under the instruction of PRISMA guidance (http://www.prisma-statement.org), and the protocol for the research was registered at PROSPERO (CRD42022343218).

### Study inclusion criteria and outcome measurements

We searched PubMed, the Web of Science, Embase, and the Cochrane Library from inception to April 2023, to identify comparative trials that compared the effectiveness and/or safety of antibiotics prophylaxis with placebo in adult patients (18 years or older) undergoing IMV in ICUs. Only the articles with an English abstract were screened. The titles and abstracts were assessed for eligibility, and the full text of studies deemed potentially relevant were reviewed. Observational and interventional studies were included if the study provided data on at least one of the following outcomes: (1) incidence of VAP; (2) mortality; (3) duration of ICU and hospital stays; and (4) duration of IMV (details are provided in the Additional files). We also checked the reference lists of the relevant articles to identify additional studies. The search was repeated before the final analyses, to review the latest studies.

### Data extraction and study quality assessment

Two investigators performed independent data extraction and analyses, with the third investigators assisting in case of discrepancies. The investigators first screened the titles and abstracts of the initial citations to exclude in vitro, animal, and pharmacokinetic studies, and protocol papers. Studies focusing on preterm neonates or pediatric patients were also excluded. The remaining articles were subsequently confirmed as eligible if they adhered to the inclusion criteria in the full text. The data of the final selected studies were extracted using predefined standardized forms including first author, publication year, study type, total number of participants, number of participants in each group, name of the specific antibiotic, and route of antibiotic administration. The outcome measures were the incidence of VAP, mortality, duration of IMV, and duration of hospitalization and ICU stay.

Randomized clinical trials included in the final analyses were scored using the risk-of-bias tool recommended by the Cochrane Collaboration, while the observational studies were evaluated using the Newcastle–Ottawa Scale score (More details are provided in Additional file 1).

### Statistical analysis

We used the Review Manager V.5.3 software (Cochrane Collaboration, Oxford, UK) for pairwise meta-analysis to calculate the risk ratio (RR) and the 95% confidence interval (95% CI) for dichotomous outcomes (mortality and adverse events), and standardized mean differences (SMDs) and 95% CI for continuous variables. When SMDs were not reported, we calculated the SMDs from other measures reported in the study; for example, standard error, t-statistics, and p values, according to Altman. Heterogeneity was assessed using the Cochran Q statistic and the I^2^ statistic, funnel plots, and subgroup analyses. The Grading of Recommendations Assessment, Development and Evaluation (GRADE) method was used to grade the quality or certainty of the outcomes and the strength of the recommendations [8].

We further performed NMA within a Bayesian framework using JAGS (version 4.3.0), R software (version 3.6.1), and the rjags and gemtc packages. To derive the incidence of VAP, the probability that each preventive regimen would be the best among all the preventive strategies was determined by evaluating the rank probabilities. A higher probability of achieving rank = 1 indicated a higher probability of that strategy being the best.

## Results

### Selection and characteristics of the studies

**Fig. 1:**
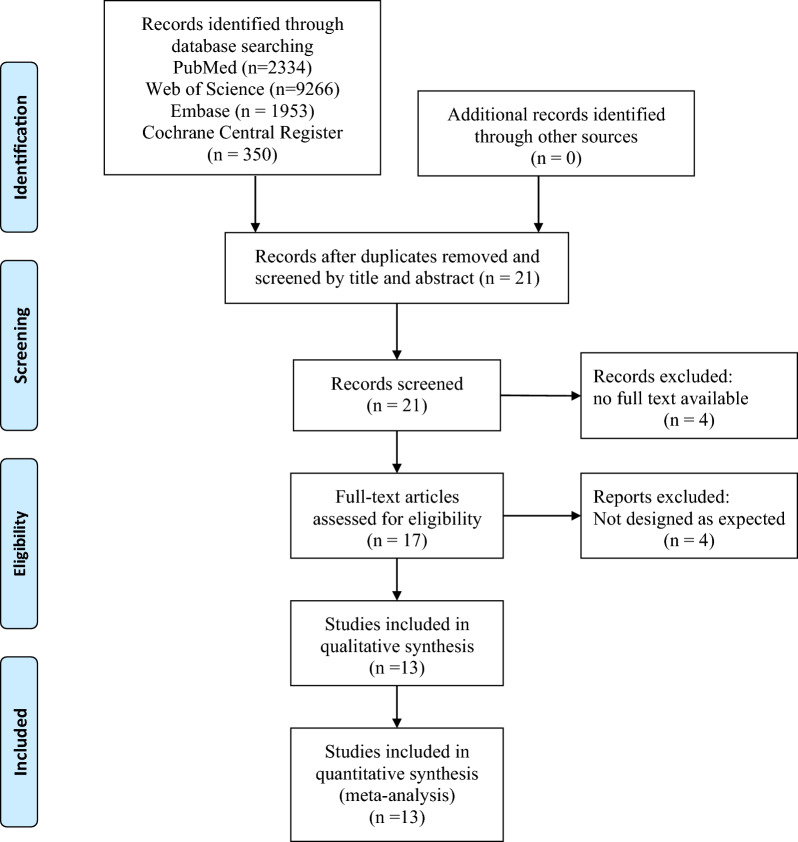
The PRISMA flow diagram

The PRISMA flow diagram is shown in Fig. 1. We initially screened 7350 articles. From these articles, we then identified 21 highly relevant articles by searching titles and abstracts and eliminating repetitions. After examining the content further, 13 studies comprising 1819 patients remained. Out of those 13 studies, 9 were randomized clinical trials, and 4 were prospective or retrospective cohort studies. The major characteristics of the included studies are shown in Table 1. The quality assessment is shown in Additional file 2: Table S1 and Figure S1.

**Table 1:** Characteristics of studies included in the meta-analysis

First author	Year	Study type	Patient population	Sample size	Antibiotics arm	Placebo arm	Intervention regimen	Administration route	Quality of study	
Karvouniaris Marios [9]	2015	Double-blind RCT	Age > 18years and have received MV > 48h	168	84	84	Nebulizing colistin 5000U thrice daily for 10 days	IH		Some concerns
Claridge Jeffrey-A [10]	2007	Double-blind RCT	MV patients with high risk for VAP	105	53	52	Nebulizing ceftazidime 250mg every 12h for seven days	IH		Low risk
Wood G-C [11]	2002	Double-blind RCT	Patients were expected to require MV more than 7 days and with high risk of VAP	40	20	20	Nebulizing ceftazidime 250mg every 12h for seven days	IH		Low risk
Klastersky J [12]	1974	Open RCT	Patients received tracheostomy	85	43	42	Endotracheal injection of gentamicin 80mg thrice daily	IH		Some concerns
H.Lode [13]	1992	Double-blind RCT	Patients under MV for more than four days	162	85	77	Endotracheal injection of gentamicin 40mg four times daily until extubation	IH		Some concerns
Francois B [14]	2019	Double-blind RCT	Age > 18 years and received MV after out-of-hospital cardiac arrest and treated with targeted temperature management	198	99	99	Intravenous Amoxicillin–clavulanate (at dose of 1g and 200mg) thrice daily for 2 days	IV		Low risk
Sirvent J-M [15]	1997	Open RCT	Patients with head injury or coma, with GCS ≤ 12, and have received MV > 72h	100	50	50	Intravenous cefuroxime 1500mg every 12h for 3.5 ± 1.8 days	IV		Some concerns
Acquarolo A [16]	2005	Open RCT	Age ≥ 18 years and comatose patients under MV, GCS ≤ 8	38	19	19	Intravenous ampicillin–sulbactam 3g every 6 h for 3 days	IV		Some concerns
Mirtalaei N [17]	2019	RCT	Stroke patients with age ≥ 20 and on MV, with GCS ≤ 8	84	42	42	Intravenous piperacillin–tazobactam 4/0.5g at intubation and 12 h later	IV		Low risk
Rouby J-J [18]	1994	Prospective non-randomized study	Patients have received MV > 72 h	598	347	251	Intratracheal injection of colistin 200,000U every 3 h for 15 days	IH		8
Rathgeber J [19]	1993	Observational study	Patients under MV > 4 days	69	29	40	Intratracheal injection of tobramycin 80mg every 6 h from intubation to extubation	IH		8
Lewis Timothy-D [20]	2018	Observational study	Age ≥ 18 and have received MV ≥ 72 h	172	81	91	Intravenous ceftriaxone 2g for single dose	IV		8
Valles J [21]	2013	Observational study	Comatose patients under MV, with GCS ≤ 8	129	71	58	Intravenous ceftriaxone 2g for single dose	IV		9

IV: drug administration via intravenous route. IH: drug administration via inhaled route

a: Quality of the observational studies was evaluated using the NOS score, while randomized studies trials using the risk-of-bias tool recommended by the Cochrane Collaboration

## Pairwise meta-analysis

### Incidence of VAP

**Fig. 2:**
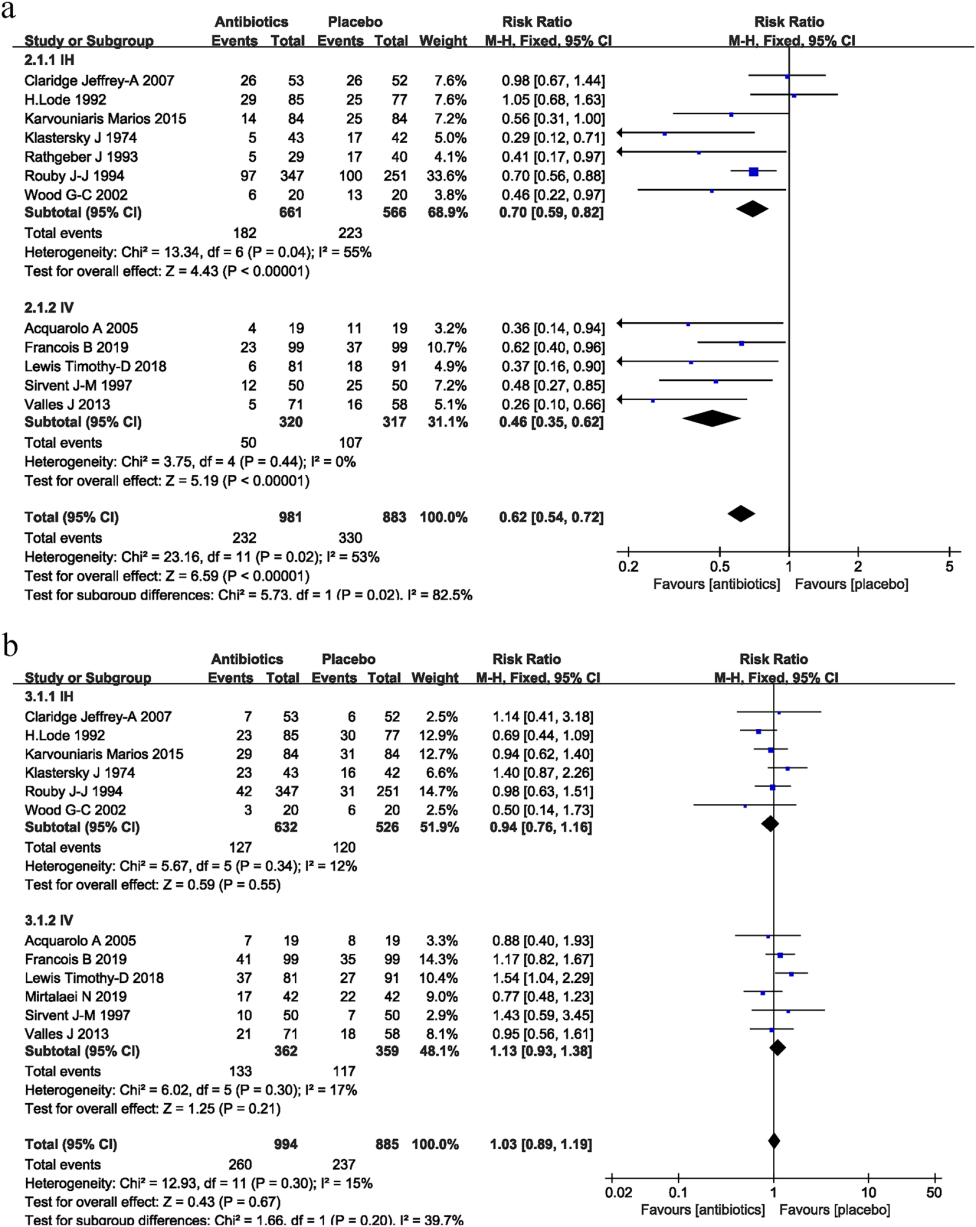
Forest plots of the effect of prophylactic antibiotics, compared with placebo, on the incidence of VAP (**A**) and mortality (**B**). Studies are grouped by the route of administration. *IV* drug administration via intravenous route. *IH* drug administration via inhaled route

The incidence of VAP in the antibiotic prophylaxis groups versus the control groups is shown in Fig. 2A. A total of 12 studies, including 1864 patients, reported the incidence of VAP. The pooled results showed that groups that received prophylactic antibiotics had a lower VAP rate compared with the control groups (RR = 0.62; 95% CI 0.54–0.72; *P* < 0.001; *I*^2^ = 53%). Of these 12 studies, 7 studies (1227 patients) reported the protective effect of prophylactic antibiotics provided through the airway, versus placebo (RR = 0.70; 95% CI 0.59–0.82; *P* < 0.001; *I*^2^ = 55%). The other five studies (637 patients) reported the beneficial effect of prophylactic antibiotics by intravenous infusion versus placebo, with no evidence of statistical heterogeneity (RR = 0.46; 95% CI 0.35–0.62; *P* < 0.0001; *I*^2^ = 0%).

### Mortality

Twelve studies, including 1879 patients, reported on mortality. There was a similar mortality between patients receiving prophylactic antibiotics and control groups (RR = 1.03; 95% CI 0.89–1.19; *P* = 0.67; *I*^2^ = 15%; Fig. 2B). In the subgroup analysis, neither antibiotics administrated via the veins (RR = 1.13; 95% CI 0.93–1.38; *P* = 0.21; *I*^2^ = 17%) nor via the respiratory tract (RR = 0.94; 95% CI 0.76–1.16; *P* = 0.55; *I*^2^ = 12%) presented a beneficial effect relative to placebo.

### Duration of IMV and ICU and hospital stays

Eight studies, including 1261 patients, reported the duration of IMV as an outcome. Both endotracheal and intravenous prophylactic antibiotics significantly reduced the duration of invasive ventilation in patients (MD =  − 2.28; 95% CI − 3.42 to 1.13; *P* < 0.0001; *I*^2^ = 72%; Additional file 2: Figure S2). Nine studies, including 1034 patients, showed a significantly shorter duration of ICU stay in the intervention group (MD =  − 1.72; 95% CI − 2.77 to − 0.67; *P* = 0.001; *I*^2^ = 24%; Additional file 2: Figure S3) compared with the control group, although the positive effect was only present in the intravenous group. Six studies of 758 patients showed no protective effect of prophylactic antibiotics on the duration of hospital stay (MD =  − 1.31; 95% CI − 3.72 to 1.11; *P* = 0.29; *I*^2^ = 37%; Additional file 2: Figure S4).

## Publication bias and sensitivity analyses

Funnel plot analysis for the incidence of VAP presented a relatively symmetric inverted plot (Additional file 2: Figure S5), indicating a publication bias for intravenous antimicrobial prophylaxis. Sensitivity analyses were also conducted to assess the impact of each study on the pooled RR; the statistical results were not markedly altered after removing any study (Additional file 2: Figure S6). The GRADE assessment showed that the quality of evidence of the results was moderate (Additional file 2: Figure S7).

## Network meta-analysis

### Network diagrams of the comparison of the incidence of VAP

**Fig. 3:**
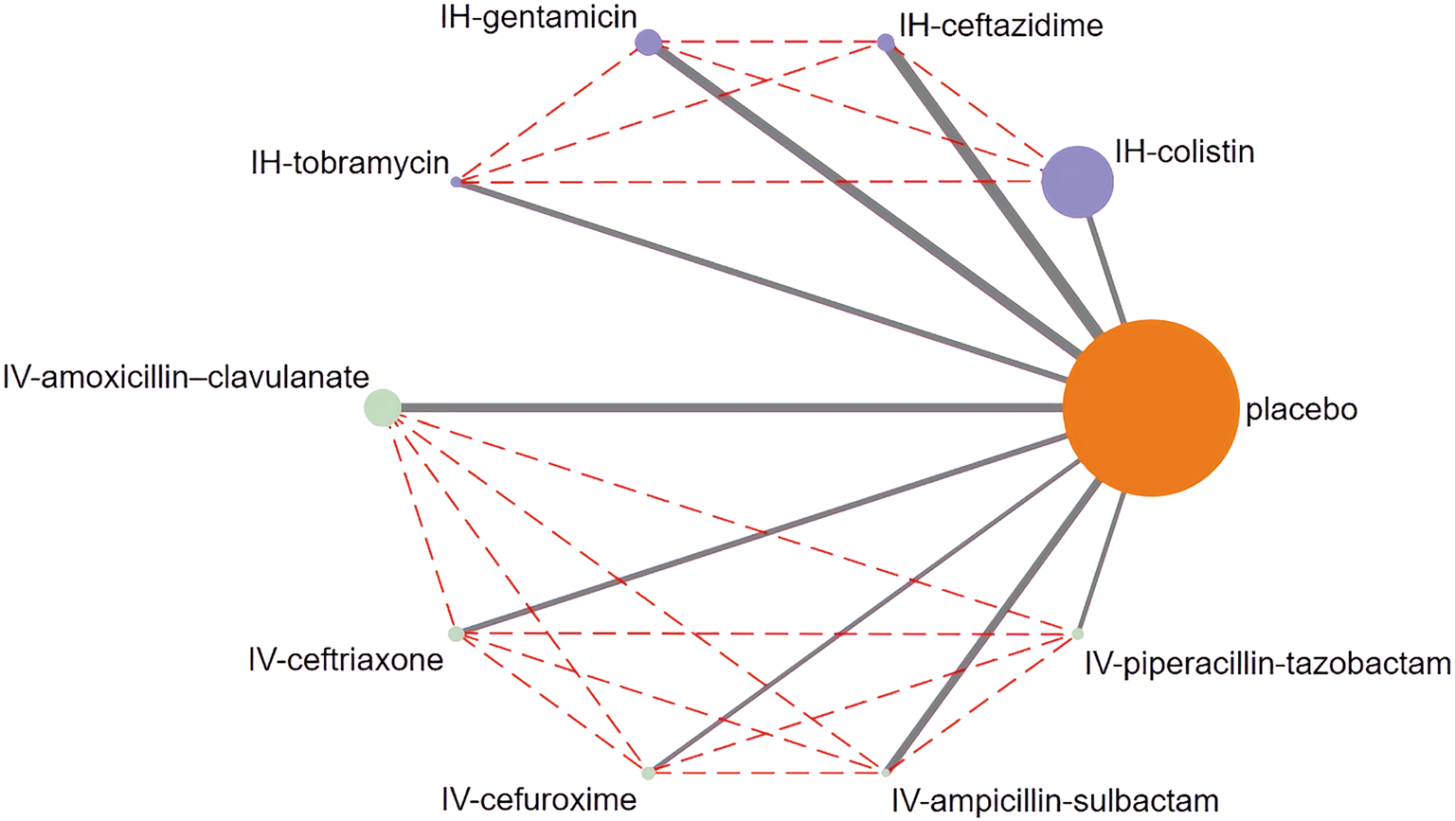
Network evidence for antibiotics to prevent VAP. *IV* drug administration via intravenous route. *IH* drug administration via inhaled route

We showed that both intravenous and inhaled antibiotics were likely associated with a reduced prevalence of VAP. However, the most protective method remained unclear because of the lack of head-to-head trials comparing different treatment strategies regardless of administration route and type of antibiotic. Thus, we conducted an NMA to obtain direct comparisons between the various strategies (Fig. 3).

### Comparison of the effect of the administration route on the incidence of VAP

**Fig. 4:**
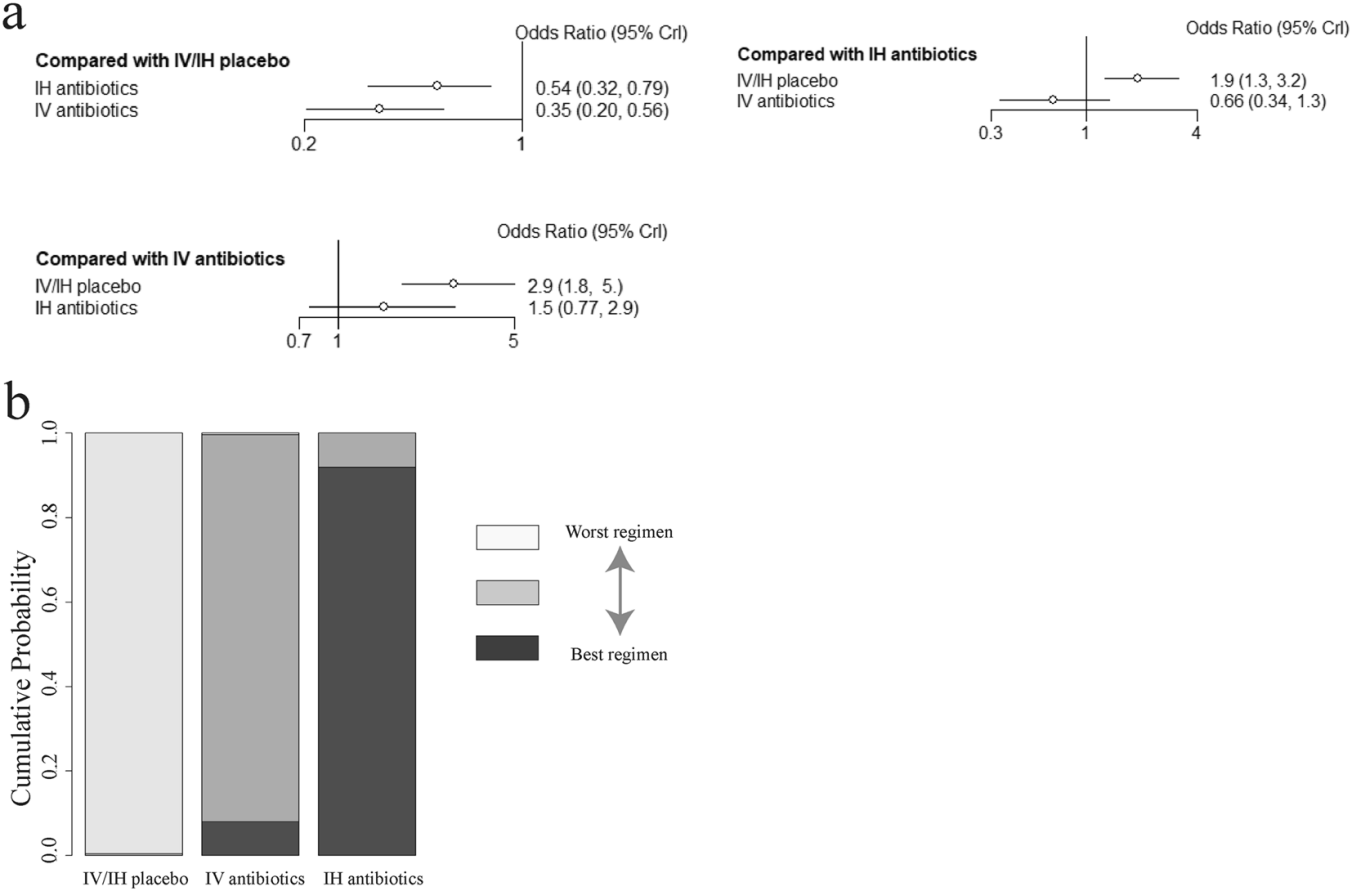
**a** Network estimates the incidence of VAP for antibiotics delivered via different administration routes. **b** Rank probabilities of the prophylactic antibiotics for the incidence of VAP based on the network meta-analysis. *CI* confidence interval. *IV* drug administration via intravenous route. *IH* drug administration via inhaled route

First, an indirect comparison between antibiotics administrated via the airway tract and intravenously was obtained, which showed that there was a lower risk of VAP in the intravenous group, but the result was not statistically significant (OR = 0.66; 95% CI 0.34–1.3). From the available data, intravenous antibiotics (91.9% probability) presented a greater likelihood of reducing the incidence of VAP than did intratracheal/inhaled antibiotics (8.03% probability) (Fig. 4).

### Comparisons between antibiotics administered the respiratory tract

**Fig. 5:**
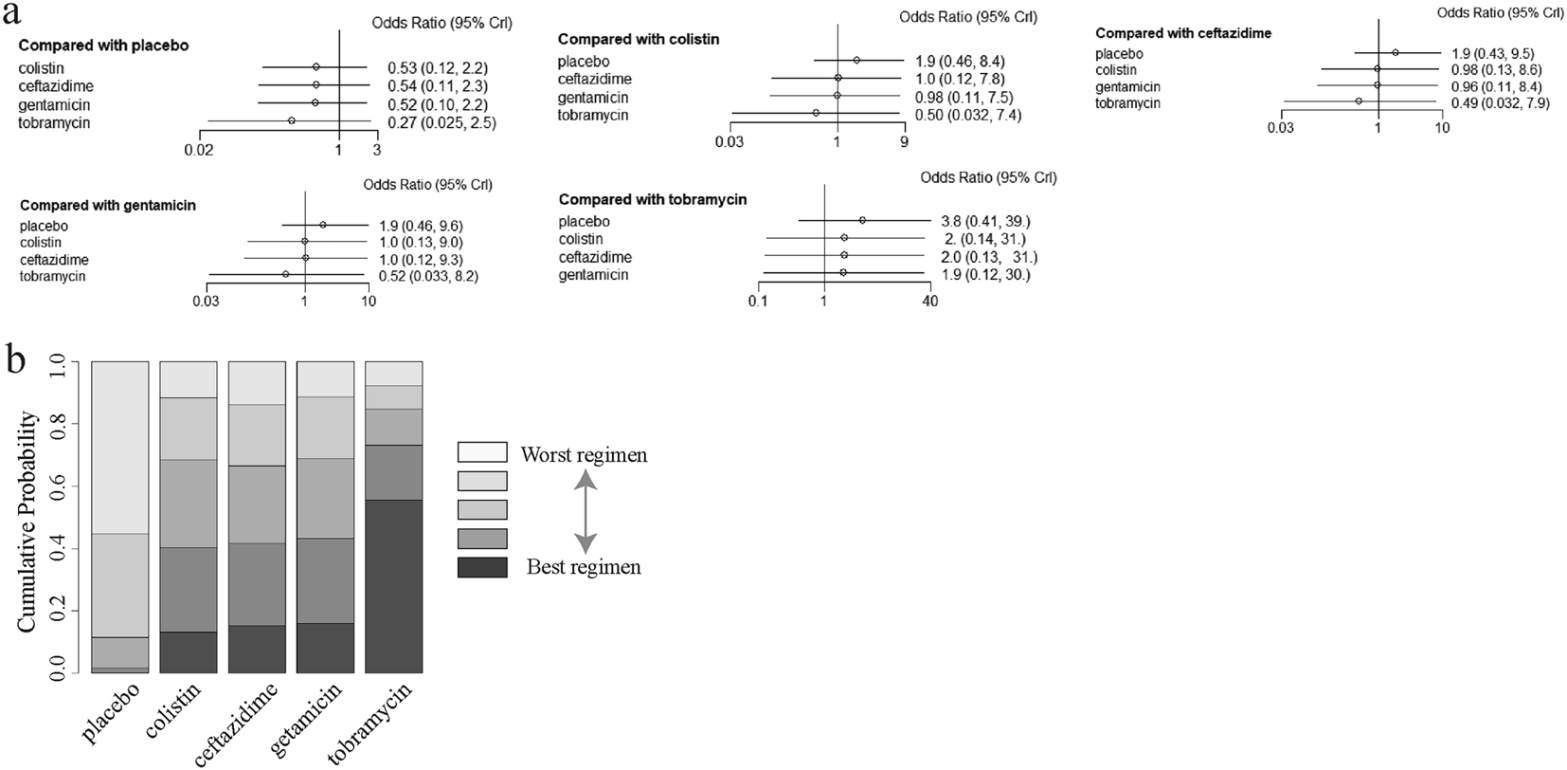
**a** Network estimates of the incidence of VAP for different antibiotics delivered via the airway tract. **b** Rank probabilities of aerosolized antibiotics delivered via the airway tract for the incidence of VAP based on the network meta-analysis. *CI* confidence interval. *IV* drug administration via intravenous route. *IH* drug administration via inhaled route

Eight trials, assessing the effect of the prophylactic antibiotics given administered the respiratory tract, were then pooled to obtain indirect comparisons by comparing aerosolized colistin, aerosolized gentamicin, aerosolized tobramycin, aerosolized ceftazidime, and aerosolized placebo one by one. In the individual comparisons, no statistically significant difference was found between each group (Fig. 5a). The assessment of rank probabilities indicated that aerosolized tobramycin (55.6% probability) presented the greatest likelihood of reducing the incidence of VAP of the four antibiotics, followed by gentamicin (15.9% probability), ceftazidime (15.3% probability), and colistin (13.1% probability) (Fig. 5b).

### Comparisons between antibiotics administered intravenously

**Fig. 6:**
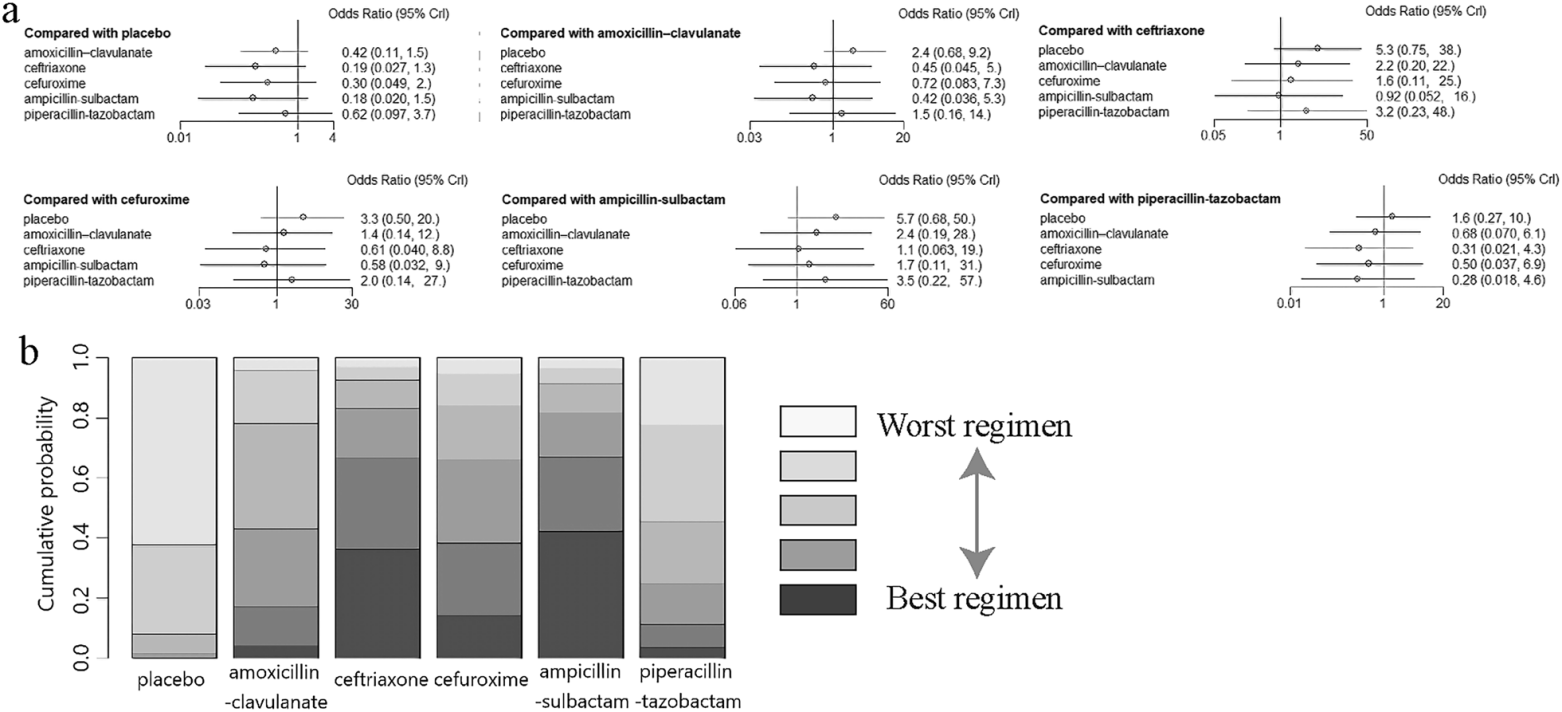
**a** Network estimates of the incidence of VAP for different antibiotics delivered via intravenous administration. **b** Rank probabilities of the aerosolized antibiotics delivered via intravenous administration for the incidence of VAP based on the network meta-analysis. *CI* confidence interval. *IV* drug administration via intravenous route. *IH* drug administration via inhaled route

Finally, five trials assessing intravenous antibiotics were pooled for indirect comparisons by comparing intravenous amoxicillin–clavulanate, cefuroxime, ampicillin–sulbactam, piperacillin–tazobactam, ceftriaxone, and placebo one by one. No statistically significant difference was found between each group (Fig. 6a). Ranking analysis indicated that ampicillin–sulbactam (42.2% probability) showed the greatest effectiveness in lowering the rate of VAP, followed by ceftriaxone (36.3%), cefuroxime (14.0%), amoxicillin–clavulanate (3.9%), and piperacillin–tazobactam (3.4%) (Fig. 6b).

## Discussion

This meta-analysis of 13 studies, which included 1819 patients requiring IMV in ICU, demonstrated that prophylactic antibiotics were associated with a reduced incidence of VAP but similar mortality, compared with placebo. The further NMA indicated that antibiotic prophylaxis via intravenous administration presented as slightly more effective in preventing VAP than administration via the respiratory tract, but the difference was not significant (OR = 0.66, 95% CI 0.34–1.3). Moreover, we found that tobramycin may be the most effective antibiotic against VAP administered via the respiratory tract (55.6% probability), and ampicillin–sulbactam may be the most effective intravenous antibiotic (42.2% probability). Another benefit of prophylactic antibiotic use was shorter durations of IMV and time in an ICU.

This is the first NMA to assess the efficacy of antibiotic prophylaxis for preventing VAP in patients undergoing IMV, including providing insights to determine the most effective antibiotics based on the NMA. Our results were consistent with the results of Póvoa et al. and Falagas et al. [6, 22], who performed meta-analyses focusing on the effect of antibiotic prophylaxis administered via the respiratory tract, and both the analyses demonstrated a protective effect of antibiotic prophylaxis. Similarly, Badve et al. and Righy et al. [23, 24] found that there was a protective effect of preventive antibiotic via intravenously route on reducing the risk of VAP in a post-stroke and comatose population. Different from the studies mentioned above, in the present meta-analysis, we focused only on those IMV patients in ICU, but did not restrict the patients’ etiology or the administration route, in an attempt to provide a more comprehensive view of the available evidence regarding preventive antibiotic use. Moreover, a further NMA was conducted to obtain an indirect comparison between different treatment strategies, and to help to determine the best recommendation for the antibiotic type and administration route.

Our results contrasted with the meta-analysis conducted by Couper et al. [25], which demonstrated no difference in the incidence of pneumonia between the preventive antibiotic group and the control group. Some factors may explain this difference. First, the population selected in Couper et al.’s study was patients after cardiac arrest. A considerable rate of these patients was receiving targeted temperature management and the lower body temperatures may have reduced inflammatory activation, and therefore, a reduced rate of VAP was detected even in the controlled group. Second, the post-arrest population may present a lower risk of infection, compared with the population in the present study that included some comatose patients at high risk of aspiration. Third, a caution was put on the evidence quality, because only three randomized clinical trials were included in Couper study.

We found that intravenous and aerosolized antibiotic had a comparable effectiveness against VAP. Intravenous administration has been considered to be the most effective treatment route, with the highest rate of drug absorption. Until now, intravenous antibiotic have been the most common approach in the treatment of VAP, and only after failure of intravenous antibiotics is the addition of inhaled antibiotics considered. However, treatment with aerosolized antibiotics is a more promising strategy than intravenous antibiotics, because aerosolized antibiotic theoretically provides a higher level of drug concentration in the lungs and a lower systematic concentration [26, 27]. Therefore, aerosolized antibiotics can contribute to a reduction of infected biofilm on the inner surface of the endotracheal tube and subsequently decrease the colonization of bacterial pathogens [28, 29]. Thus, the intravenous and inhaled route are both acceptable and the choice of route should be depended on the clinical situation.

Furthermore, by NMA modeling, we found that tobramycin via nebulization and ampicillin–sulbactam via intravenous administration presented the greatest probabilities of being the most efficient regimens for preventing VAP. This result may be explained by the fact that most of the included studies focused on early onset VAP, defined as happening within the first 4–7 days of hospitalization, which was more likely to be caused by bacteria sensitive to ampicillin–sulbactam and tobramycin [30]. Nebulization of tobramycin has frequently been clinically validated in the treatment of bronchiectasis patients infected with *Pseudomonas aeruginosa* and has been approved by the Food and Drug Administration [31]. However, considering the small sample size in the relevant study [19], the conclusions should be reviewed cautiously and further evaluated in the future.

Theoretically, because the application of antibiotic prophylaxis reduced the incidence of VAP, it should be associated with a reduction in mortality. However, no reduction in mortality was found, from the first study in 1974 through to the latest study in 2019, either via respiratory-tract or intravenous administration. Several factors may explain these findings. First, VAP is only responsible for some of the deaths in ICUs. For example, 82% of those receiving targeted temperature management after cardiac arrest died of cardiac or neurological failure, whereas only 12% of patients died from infections that might have benefited from antibiotic prophylaxis [32]. Second, the insufficient sample sizes and/or the limited extent of the VAP rate reduction might be reflected in the lack of effect on the mortality. Third, most of the studies that we included focused on the incidence of early onset VAP, which is caused by less virulent microorganisms and causes less mortality. In a study that compared the different impacts on mortality of early and late VAP, late VAP was associated with higher ICU mortality. In addition, we found that there was an approximately 2-day reduction in the duration of the ICU stay or IMV for patients receiving prophylactic antibiotics, which may be attributed to the reduction in the incidence of VAP, and which represented a likelihood of reduced economic burden.

Some limitations existed in the present study. First, because the emergency of multi-drug resistant bacteria (MDRB) was not considered in most of the included studies, we did not obtain sufficient data to assess the risk of MDRB between the intervention and control groups. However, MDRB was not a major component in the present study and none of the included studies presented a different risk of MDRB between groups. Second, there was a substantial timespan from the first to the most recent study; therefore, there was a high possibility that changes in clinical practice had occurred over this time. For example, intratracheal instillation is no longer performed, whereas nebulization techniques have improved considerably [33, 34]. However, this is an unavoidable problem for such a research topic with few clinical trials. Moreover, gentamicin (delivered via instillation) did not show an apparent difference in the rate of VAP compared with ceftazidime or colistin (delivered via nebulization mostly). Thus, the type of nebulization technique may not have had a considerable impact on our results. Third, the heterogeneous design and various underlying diseases of patients recruited in included studies may be another confounding factor. However, most included studies recruiting traumatic, postsurgical or comatose patients, rather than those with pre-existing respiratory-tract infection. Moreover, some studies were non-RCT, but sensitivity analysis showed that they imposed no significant effect of overall result. Therefore, this confounding factor was acceptable for our research objective. At last, NMA only provided indirect comparison between different drugs using a blank control (Rouby J-J, 1994) and saline group as reference. The potential therapeutic effects saline can cause an impact on the study results to some certain extent. However, in our sensitivity analysis, excluding the results of Rouby J–J’s study did not significantly affect the final results. Therefore, we believed that the therapeutic effect of saline has a minimal impact on the overall results. However, we still need head-to-head RCTs to provide more strong evidences.

## Conclusions

According to our results, antibiotic prophylaxis may reduce the incidence of VAP, but not reduce mortality, for adult patients receiving IMV in ICUs; an NMA demonstrated that tobramycin via nebulization and ampicillin–sulbactam via intravenous administration presented the greatest possibility of being the most effective regimens for preventing VAP. However, it should be highlighted that, because of the low level of evidence of most of the included studies, we cannot make any strong suggestions until additional, well-designed randomized studies with large sample sizes are conducted.

### Supplementary Information

**Additional file 1: Methods. **Search strategy, study inclusion criteria and outcome measurements, quality assessment and statistical analysis.

**Additional file 2: Table S1**. The quality assessments of observational studies using Newcastle-Ottawa Scale score. **Figure S1**. The quality assessments of interventional studies using risk of bias tool 2 （ROB2）recommended by the Cochrane Collaboration. **Figure S2.** Forest plots of the effect of prophylactic antibiotics compared with placebo, on the duration of invasive ventilation. Studies are grouped by the routes of administration. **Figure S3.** Forest plots of the effect of prophylactic antibiotics, compared with placebo, on the duration of ICU stay. Studies are grouped by the route of administration. **Figure S4.** Forest plots of the effect of prophylactic antibiotics compared with placebo, on the duration of hospitalization. Studies are grouped by the route of administration. **Figure S5.** Forest plots of the effect of prophylactic antibiotics, compared with placebo, on adverse events. Studies are grouped by the route of administration. **Figure S6.** Funnel plot of the included studies for incidence of VAP. **Figure S7.** Sensitivity analyses on the incidence of VAP. **Figure S8.** GRADE assessment of a) randomized controlled studies and b) observational studies.

## Data Availability

All data generated or analyzed during this study are included in this published article and its supplementary information files.

## Abbreviations

VAP: Ventilator-associated pneumonia
IMV: Invasive mechanical ventilation
ICU: Intensive-care unit
NMA: Network meta-analysis
RR: Risk ratio
SMDs: Standardized mean differences
GRADE: Grading of Recommendations Assessment, Development and Evaluation
MDRB: Multi-drug resistant bacterial

## References

[1] Rosenthal VD, Bijie H, Maki DG (2012). International nosocomial infection control consortium (INICC) report, data summary of 36 countries, for 2004–2009. Am J Infect Control.

[2] Papazian L, Klompas M, Luyt C (2020). Ventilator-associated pneumonia in adults: a narrative review. Intensive Care Med.

[3] Kalil AC, Metersky ML, Klompas M (2016). Management of adults with hospital-acquired and ventilator-associated pneumonia: 2016 clinical practice guidelines by the infectious diseases society of America and the American thoracic society. Clin Infect Dis.

[4] Klompas M, Branson R, Eichenwald EC (2014). Strategies to prevent ventilator-associated pneumonia in acute care hospitals: 2014 update. Infect Control Hosp Epidemiol.

[5] Nolan JP, Sandroni C, Bottiger BW (2021). European resuscitation council and European society of intensive care medicine guidelines 2021: post-resuscitation care. Intensive Care Med.

[6] Póvoa FCC, Cardinal-Fernandez P, Maia IS (2018). Effect of antibiotics administered via the respiratory tract in the prevention of ventilator-associated pneumonia: a systematic review and meta-analysis. J Crit Care.

[7] Cipriani A, Higgins JP, Geddes JR (2013). Conceptual and technical challenges in network meta-analysis. Ann Intern Med.

[8] Atkins D, Best D, Briss PA (2004). Grading quality of evidence and strength of recommendations. BMJ.

[9] Karvouniaris M, Makris D, Zygoulis P (2015). Nebulised colistin for ventilator-associated pneumonia prevention. Eur Respir J.

[10] Claridge JA, Edwards NM, Swanson J (2007). Aerosolized ceftazidime prophylaxis against ventilator-associated pneumonia in high-risk trauma patients: results of a double-blind randomized study. Surg Infect.

[11] Wood GC, Boucher BA, Croce MA (2002). Aerosolized ceftazidime for prevention of ventilator-associated pneumonia and drug effects on the proinflammatory response in critically ill trauma patients. Pharmacotherapy.

[12] Klastersky J, Huysmans E, Weerts D (1974). Endotracheally administered gentamicin for the prevention of infections of the respiratory tract in patients with tracheostomy: a double-blind study. Chest.

[13] Lode H, Hoffken G, Kemmerich B (1992). Systemic and endotracheal antibiotic prophylaxis of nosocomial pneumonia in ICU. Intensive Care Med.

[14] Francois B, Cariou A, Clere-Jehl R (2019). Prevention of early ventilator-associated pneumonia after cardiac arrest. N Engl J Med.

[15] Sirvent JM, Torres A, El-Ebiary M (1997). Protective effect of intravenously administered cefuroxime against nosocomial pneumonia in patients with structural coma. Am J Respir Crit Care Med.

[16] Acquarolo A, Urli T, Perone G (2005). Antibiotic prophylaxis of early onset pneumonia in critically ill comatose patients. A randomized study. Intensive Care Med.

[17] Mirtalaei N, Farazi A, Ebrahimi MM (2019). Efficacy of antibiotic prophylaxis against ventilator-associated pneumonia. J Hosp Infect.

[18] Rouby JJ, Poete P, Martin DLE (1994). Prevention of gram negative nosocomial bronchopneumonia by intratracheal colistin in critically ill patients. Histologic and bacteriologic study. Intensive Care Med.

[19] Rathgeber J, Zielmann S, Panzer C (1993). Prevention of pneumonia by endotracheal micronebulization of tobramycin. Anasthesiol Intensivmed Notfallmed Schmerzther.

[20] Lewis TD, Dehne KA, Morbitzer K (2018). Influence of single-dose antibiotic prophylaxis for early-onset pneumonia in high-risk intubated patients. Neurocrit Care.

[21] Valles J, Peredo R, Burgueno MJ (2013). Efficacy of single-dose antibiotic against early-onset pneumonia in comatose patients who are ventilated. Chest.

[22] Falagas ME, Siempos II, Bliziotis IA (2006). Administration of antibiotics via the respiratory tract for the prevention of ICU-acquired pneumonia: a meta-analysis of comparative trials. Crit Care.

[23] Badve MS, Zhou Z, Anderson CS (2018). Effectiveness and safety of antibiotics for preventing pneumonia and improving outcome after acute stroke: systematic review and meta-analysis. J Stroke Cerebrovasc Dis.

[24] Righy C, Do BP, Valles J (2017). Systemic antibiotics for preventing ventilator-associated pneumonia in comatose patients: a systematic review and meta-analysis. Ann Intensive Care.

[25] Couper K, Laloo R, Field R (2019). Prophylactic antibiotic use following cardiac arrest: a systematic review and meta-analysis. Resuscitation.

[26] Le Conte P, Potel G, Peltier P (1993). Lung distribution and pharmacokinetics of aerosolized tobramycin. Am Rev Respir Dis.

[27] Odio W, Van Laer E, Klastersky J (1975). Concentrations of gentamicin in bronchial secretions after intramuscular and endotracheal administration. J Clin Pharmacol.

[28] Inglis TJ, Millar MR, Jones JG (1989). Tracheal tube biofilm as a source of bacterial colonization of the lung. J Clin Microbiol.

[29] Adair CG, Gorman SP, Feron BM (1999). Implications of endotracheal tube biofilm for ventilator-associated pneumonia. Intensive Care Med.

[30] Guidelines for the Management of Adults with Hospital-acquired (2005). Ventilator-associated, and healthcare-associated pneumonia. Am J Respir Crit Care Med.

[31] Expert consensus on nebulization therapy in pre-hospital and in-hospital emergency care. Ann Transl Med, 2019; 7(18):487. 10.21037/atm.2019.09.44 PMC6803223 31700923

[32] Nielsen N, Wetterslev J, Cronberg T (2013). Targeted temperature management at 33 degrees C versus 36 degrees C after cardiac arrest. N Engl J Med.

[33] Le J, Ashley ED, Neuhauser MM (2010). Consensus summary of aerosolized antimicrobial agents: application of guideline criteria. Insights from the society of infectious diseases pharmacists. Pharmacotherapy.

[34] Palmer LB (2009). Aerosolized antibiotics in critically ill ventilated patients. Curr Opin Crit Care.

