# Supplementary material for: Prophylactic antibiotics for preventing ventilator-associated pneumonia: a pairwise and Bayesian network meta-analysis
Source: Eur J Med Res. 2023 Sep 15;28:348. doi: 10.1186/s40001-023-01323-z (PMC10503075; doi:10.1186/s40001-023-01323-z)
Supplement: Supplementary file 1 — Additional file 1: Methods. Search strategy, study inclusion criteria and outcome measurements, quality assessment and statistical analysis. [file 40001_2023_1323_MOESM1_ESM.docx]

**Supplement materials**

**Methods**

***Search strategy***

We searched PubMed, the Web of Science, Embase and the Cochrane Library to identify potentially relevant studies from inception to December 2021, and only searched the article with English abstract. The main terms adopted were as follows: 1) ventilator-associated pneumonia OR hospital-acquired pneumonia OR nosocomial pneumonia OR VAP OR respiratory infection; 2) antibiotic OR colistin OR amikacin OR tobramycin OR ceftazidime OR cefuroxime OR polymyxin; 3) prevent* OR prophylaxis OR protect*. We also checked the reference lists of the relevant publications to identify additional studies. The search was repeated before the final analyses, to review the latest studies.

***Study inclusion criteria and outcomes measurements***

In our meta-analysis, we included those comparative trials that compared the effectiveness and/or safety of prophylaxis antibiotics with placebo in adult patients (18 years or older) under IMV in ICU. The selected studies were composed of observational and interventional studies. The final studies chosen were required to assess at least one of the following outcomes: 1) incidence of VAP(1), defined as the emergence of new or progressive radiographic pulmonary infiltrate and two of the following criteria: abnormal temperature (>38 °C or <36 °C), abnormal white blood cell count and purulent tracheobronchial aspirates with a positive culture, occurring at least 48 hours after starting IMV (in more detail, the result with the longest observation time was selected if the author recorded more than one time point); 2) mortality, defined as percentage of patients dying during the ICU period or throughout hospitalization period; 3) duration of ICU and hospital stays; 4) duration of IMV; and 5) incidence of positive bacterial cultures.

***Quality assessment***

Randomized clinical trials included in the final analyses were scored using the risk-of-bias tool recommended by the Cochrane Collaboration. Six domains were assessed: selection bias, performance bias, detection bias, attrition bias, reporting bias and other biases, with each domain being judged as ‘low risk,’ ‘unclear risk’ or ‘high risk.’ The observational studies were evaluated using the Newcastle–Ottawa Scale score, composed of three components including nine items related to the study cohort selection, comparability and outcome. Research was graded as of good quality if it scored ≥7.

***Statistical analysis***

Heterogeneity was assessed using the Cochran Q statistic and the I2 statistic. I^2^ values ≤50% were considered to indicate acceptable heterogeneity between studies and the fixed-effects model was selected. Otherwise, we identified the source of heterogeneity by subgroup analysis or sensitivity analysis, or used the random-effects model. Publication bias was determined using the funnel plots. For subgroup analyses, we split the studies into two subgroups according to their route of antibiotic used and applied a pairwise meta-analysis model separately in each subgroup.

**References:**

1. Nseir S, Favory R, Jozefowicz E, Decamps F, Dewavrin F, Brunin G, Di Pompeo C, Mathieu D, Durocher A. Antimicrobial treatment for ventilator-associated tracheobronchitis: a randomized, controlled, multicenter study. *CRIT CARE* 2008;12:R62.
