# Supplementary material for: Prophylactic antibiotics for preventing ventilator-associated pneumonia: a pairwise and Bayesian network meta-analysis
Source: Eur J Med Res. 2023 Sep 15;28:348. doi: 10.1186/s40001-023-01323-z (PMC10503075; doi:10.1186/s40001-023-01323-z)
Supplement: Supplementary file 2 — Additional file 2: Table S1. The quality assessments of observational studies using Newcastle-Ottawa Scale score. Figure S1. The quality assessments of interventional studies using risk of bias tool 2 （ROB2）recommended by the Cochrane Collaboration. Figure S2. Forest plots of the effect of prophylactic antibiotics compared with placebo, on the duration of invasive ventilation. Studies are grouped by the routes of administration. Figure S3. Forest plots of the effect of prophylactic antibiotics, compared with placebo, on the duration of ICU stay. Studies are grouped by the route of administration. Figure S4. Forest plots of the effect of prophylactic antibiotics compared with placebo, on the duration of hospitalization. Studies are grouped by the route of administration. Figure S5. Forest plots of the effect of prophylactic antibiotics, compared with placebo, on adverse events. Studies are grouped by the route of administration. Figure S6. Funnel plot of the included studies for incidence of VAP. Figure S7. Sensitivity analyses on the incidence of VAP. Figure S8. GRADE assessment of a) randomized controlled studies and b) observational studies. [file 40001_2023_1323_MOESM2_ESM.docx]

**Additional materials**

**Table S1**. The quality assessments of observational studies using Newcastle-Ottawa Scale score.


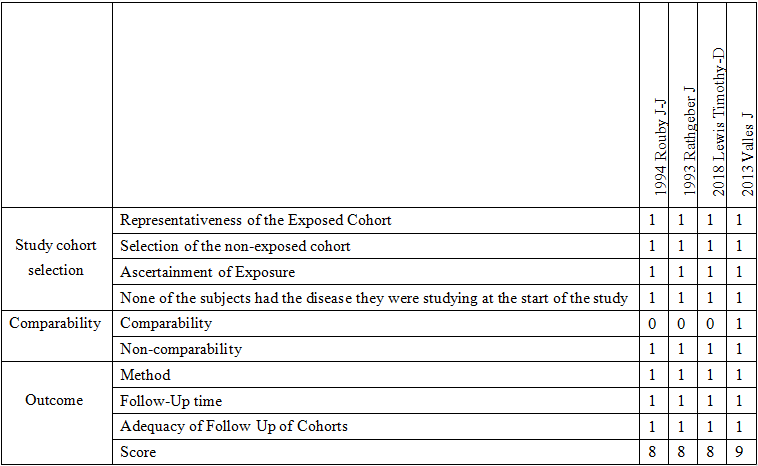


**Figure S1**. The quality assessments of interventional studies using risk of bias tool 2 （ROB2）recommended by the Cochrane Collaboration.


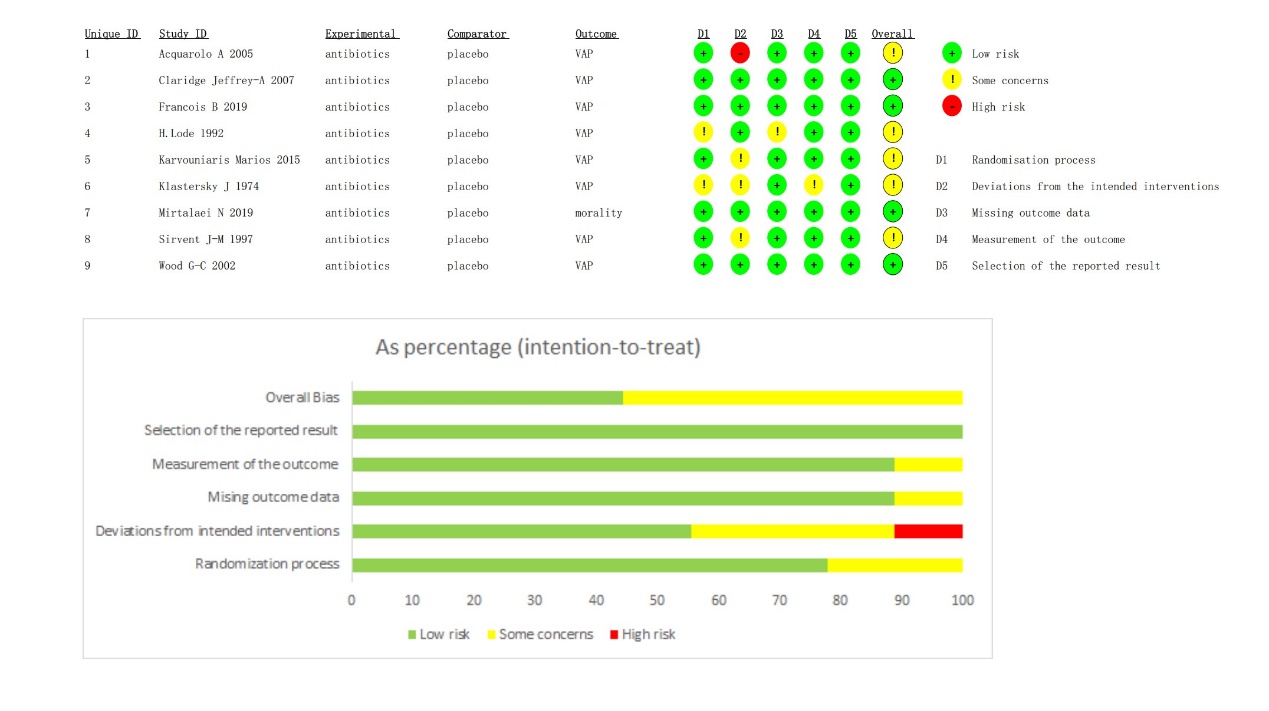


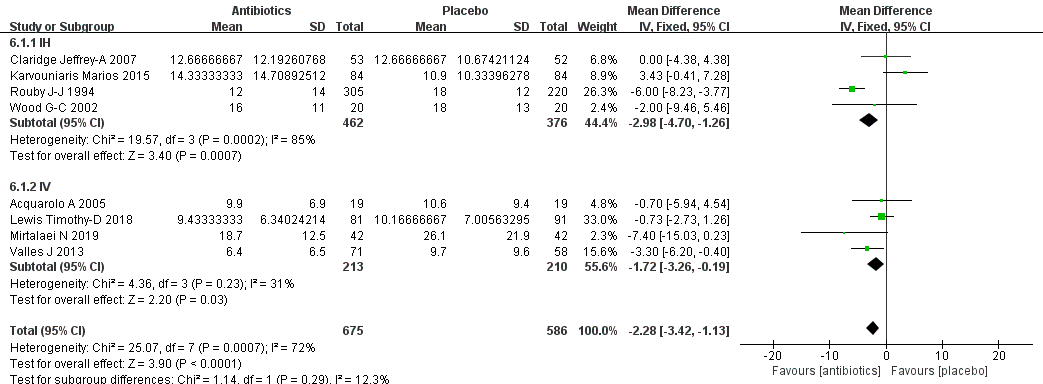


**Figure S2.** Forest plots of the effect of prophylactic antibiotics compared with placebo, on the duration of invasive ventilation. Studies are grouped by the routes of administration


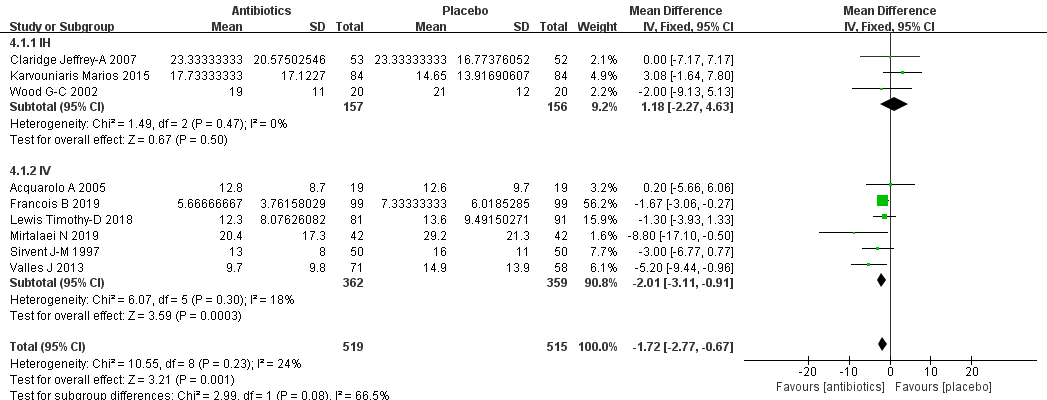


**Figure S3.** Forest plots of the effect of prophylactic antibiotics, compared with placebo, on the duration of ICU stay. Studies are grouped by the route of administration


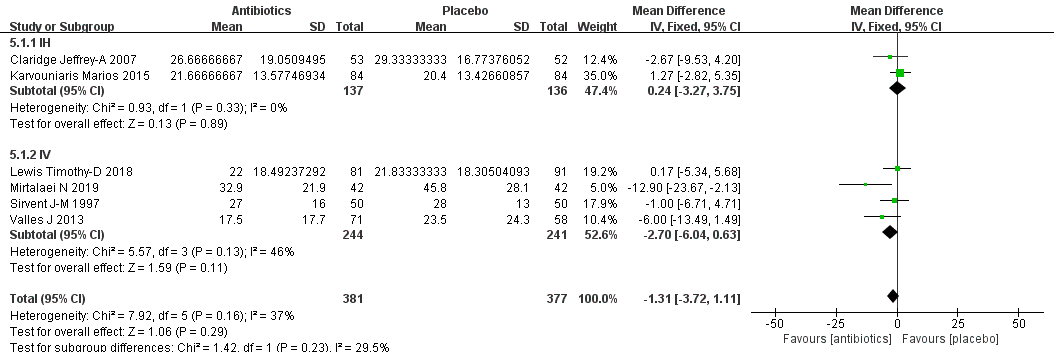


**Figure S4.** Forest plots of the effect of prophylactic antibiotics compared with placebo, on the duration of hospitalization. Studies are grouped by the route of administration.


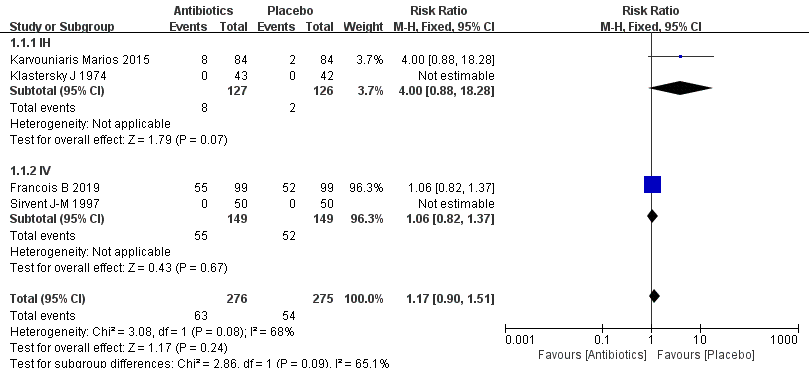


**Figure S5.** Forest plots of the effect of prophylactic antibiotics, compared with placebo, on adverse events. Studies are grouped by the route of administration.


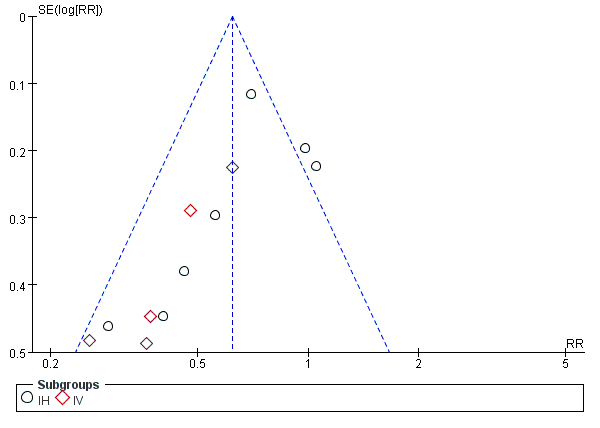


**Figure S6.** Funnel plot of the included studies for incidence of VPA.


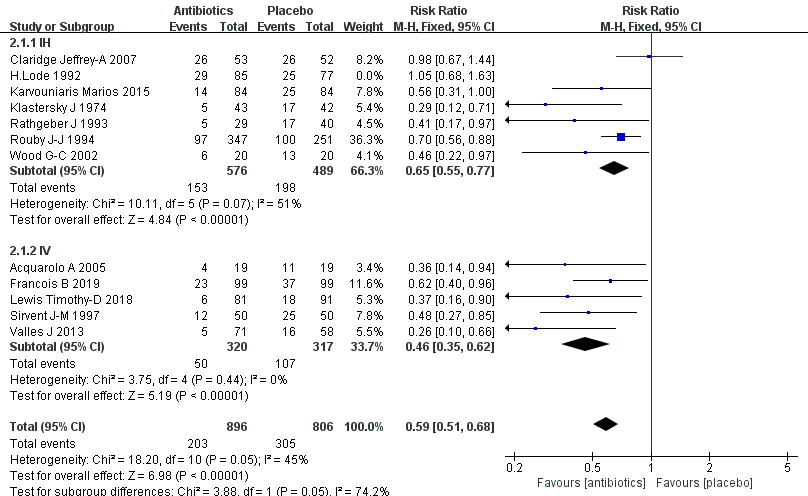


(a)


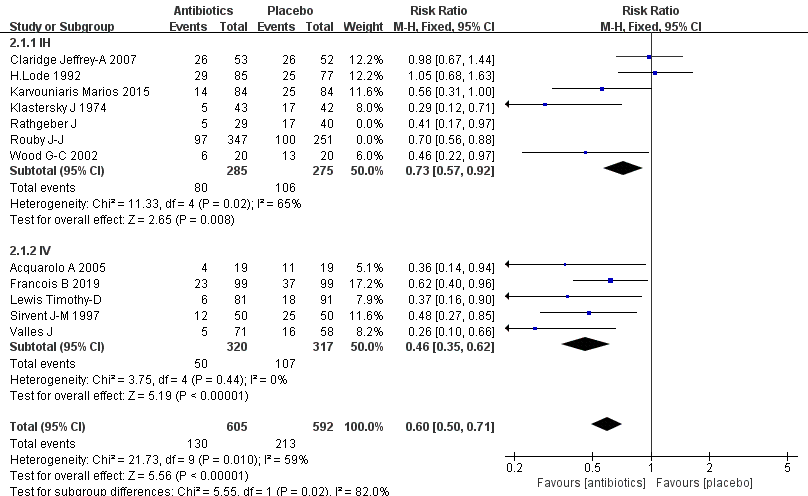


(b)

**Figure S7.** Sensitivity analyses on the incidence of VAP. (a)Removing the study of H.Lode with the highest heterogeneity. (b) Removing two non-RCT studies of Rouby J-J and Rathgeber J.


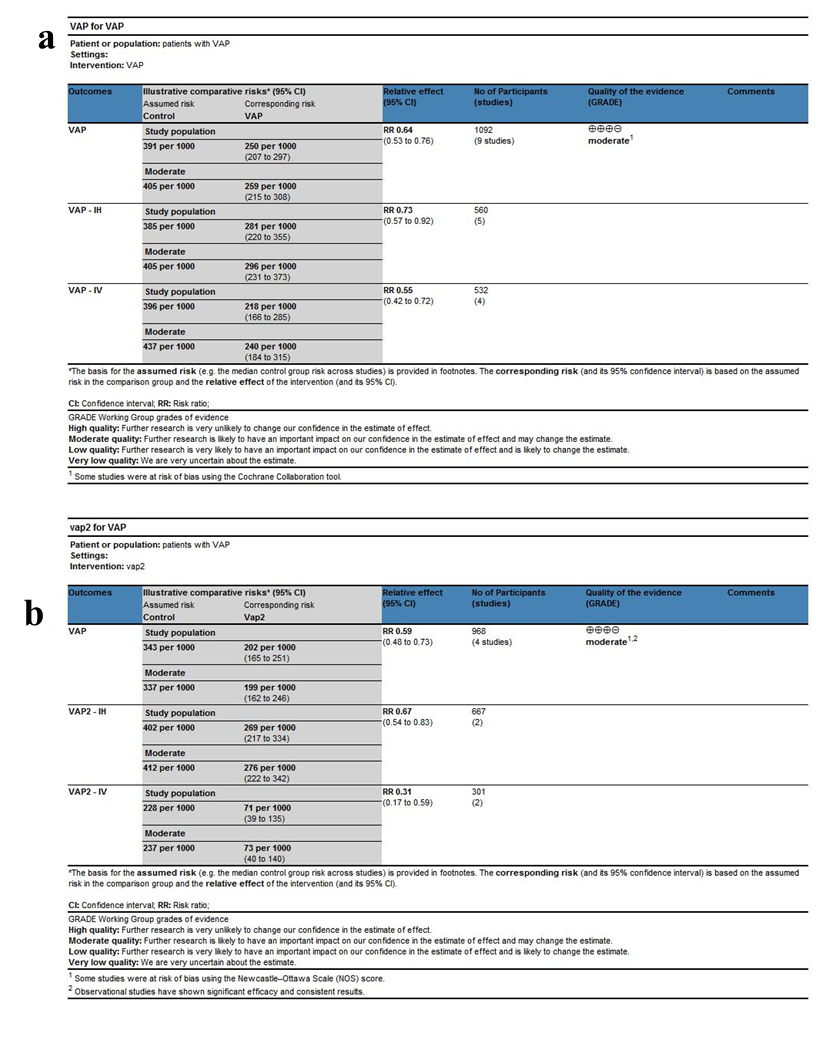


**Figure S8.** GRADE assessment of a) randomized controlled studies and b) observational studies**.**
